# Protein arginine methyltransferase 1 regulates cell proliferation and differentiation in adult mouse adult intestine

**DOI:** 10.1186/s13578-021-00627-z

**Published:** 2021-06-22

**Authors:** Lu Xue, Lingyu Bao, Julia Roediger, Yijun Su, Bingyin Shi, Yun-Bo Shi

**Affiliations:** 1 Institute for Medical Biology and Hubei Provincial Key Laboratory for Protection and Application of Special Plants in Wuling Area of China, College of Life Sciences, South-Central University for Nationalities, 182 Minyuan Road, Hongshan District, Wuhan, 430074 China; 2 Department of Endocrinology, The First Affiliated Hospital of Xi’an Jiaotong University School of Medicine, No. 277, Yanta West Road, Xi’an, 710061 Shaanxi People’s Republic of China; 3 Section on Molecular Morphogenesis, Eunice Kennedy Shriver National Institute of Child Health and Human Development (NICHD), National Institutes of Health (NIH), Bethesda, MD 20892 USA; 4 Laboratory of High Resolution Optical Imaging and Advanced Imaging and Microscopy Resource, National Institute of Biomedical Imaging and Bioengineering (NIBIB), National Institutes of Health (NIH), Bethesda, MD 20892 USA

**Keywords:** Adult organ-specific stem cell, Histone arginine methyltransferase, Intestine, Transcription coactivator, Thyroid hormone receptor

## Abstract

**Background:**

Adult stem cells play an essential role in adult organ physiology and tissue repair and regeneration. While much has been learnt about the property and function of various adult stem cells, the mechanisms of their development remain poorly understood in mammals. Earlier studies suggest that the formation of adult mouse intestinal stem cells takes place during the first few weeks after birth, the postembryonic period when plasma thyroid hormone (T3) levels are high. Furthermore, deficiency in T3 signaling leads to defects in adult mouse intestine, including reduced cell proliferation in the intestinal crypts, where stem cells reside. Our earlier studies have shown that protein arginine methyltransferase 1 (PRMT1), a T3 receptor coactivator, is highly expressed during intestinal maturation in mouse.

**Methods:**

We have analyzed the expression of PRMT1 by immunohistochemistry and studied the effect of tissue-specific knockout of PRMT1 in the intestinal epithelium.

**Results:**

We show that PRMT1 is expressed highly in the proliferating transit amplifying cells and crypt base stem cells. By using a conditional knockout mouse line, we have demonstrated that the expression of PRMT1 in the intestinal epithelium is critical for the development of the adult mouse intestine. Specific removal of PRMT1 in the intestinal epithelium results in, surprisingly, more elongated adult intestinal crypts with increased cell proliferation. In addition, epithelial cell migration along the crypt-villus axis and cell death on the villus are also increased. Furthermore, there are increased Goblet cells and reduced Paneth cells in the crypt while the number of crypt base stem cells remains unchanged.

**Conclusions:**

Our finding that PRMT1 knockout increases cell proliferation is surprising considering the role of PRMT1 in T3-signaling and the importance of T3 for intestinal development, and suggests that PRMT1 likely regulates pathways in addition to T3-signaling to affect intestinal development and/or homeostasis, thus affecting cell proliferating and epithelial turn over in the adult.

**Supplementary Information:**

The online version contains supplementary material available at 10.1186/s13578-021-00627-z.

## Introduction

Adult vertebrate organs/tissues that are exposed to the external environment, such as skin and hair, undergo self-renewal to maintain tissue homeostasis and repair damages due to the external exposure. One of the best-studied self-renewing organs is the mammalian intestine. In the adult intestine, the epithelium lines the luminal side of the crypts and villi. Throughout adult life, the intestinal epithelium is replaced once every 1–6 days in mammals [1–3]. This occurs through the proliferation of the adult intestinal stem cells localized in the crypt. As the cells migrate along the crypt-villus axis, they differentiate into various types of epithelial cells and the fully differentiated epithelial cells are removed through apoptosis, mostly near the tip of the intestinal villus. Such a rapid and compete self-renewing process has led to extensive studies on the function and properties of the adult intestinal stem cells, with many genes and signaling pathways critical for the adult stem cells discovered and characterized over the years [3, 4]. On the other hand, much less is known about how the adult stem cells are formed during mammalian development.

Early morphological and recent molecular and genetics studies indicate that the mammalian intestine mature into the adult form around birth in preparation for the change in diets. In mouse, the intestine at birth has well-developed villus but lacks any crypts [5, 6]. During the next few weeks, the inter-villus region invaginates into the underlying connective tissue and gradually develops into crypts, where adult stem cells reside and epithelial cell proliferation takes place [5, 6]. About 3 weeks after birth, the mouse intestine has the morphology of the adult intestine, i.e., the presence of villus-crypt axis [5–7] and weaning occurs.

While the exact mechanism governing this intestinal maturation is unknown, plasma thyroid hormone (T3) concentration is known to rise after birth and peaks around 2–3 weeks later in mouse [8]. Furthermore, mice with T3 deficiency and/or altered T3 receptor (TR) level or function have abnormal intestinal morphology and reduced cell proliferation in the intestine [9–14], indicating that T3 is critical for intestinal development and/or maintenance of the adult intestinal physiology.

T3 can regulate target gene transcription through TRs [15–18]. For T3-inducible genes, TRs form heterodimers with 9-cis retinoic acid receptors (RXRs) and bind to T3 response elements (TREs) in the target genes to regulate their transcription in a T3-dependent manner by recruiting different cofactor complexes [16, 18–23]. Among the coactivators that are recruited by liganded TR to target genes is protein arginine methyltransferase 1 (PRMT1) [24, 25]. PRMT1 can directly methylate R3 residue of histone H4 [26, 27] and indirectly affect histone H3 and H4 acetylation and histone H3 R17 methylation [28–30], thus facilitating the formation of transcriptionally active chromatin.

Interestingly, we have shown previously that high levels of PRMT1 mRNA are expressed in an evolutionally conserved manner during the formation of the adult intestine in vertebrates, including intestinal maturation in mouse around 2–3 weeks after birth and intestinal remodeling during *Xenopus laevis* metamorphosis [31]. Importantly, intestinal remodeling during amphibian metamorphosis involves de novo development of adult stem cells in a T3-dependent process and that T3 signaling in the larval intestine is both necessary and sufficient for the formation of the adult stem cells through the dedifferentiation of some larval epithelial cells [32–40]. Furthermore, transgenic and knockdown studies in *Xenopus* have revealed a critical role of PRMT1 as a TR coactivator for amphibian metamorphosis and in the development of the adult stem cells [31].

It has been more difficult to study the role of endogenous PRMT1 in the development of adult intestine with gene knockout approaches as total knockout of PRMT1 lead to embryonic lethality in mouse and premetamorphic tadpole lethality in *Xenopus* [41, 42]. Here we have investigated the role of PRMT1 in mouse intestine by knocking out PRMT1 specifically in the intestinal epithelium. We show that intestinal specific deletion of PRMT1 has no overt effect on mouse development. On the other hand, the adult intestine has altered intestinal crypt morphology and surprisingly increased cell proliferation in the crypts and apoptosis on the villus. Our results suggest that PRMT1 plays a critical role in the maturation of the intestine and/or maintenance of the adult intestine, likely through a function other than as a TR coactivator.

## Materials and methods

### Animals

**Fig. 1:**
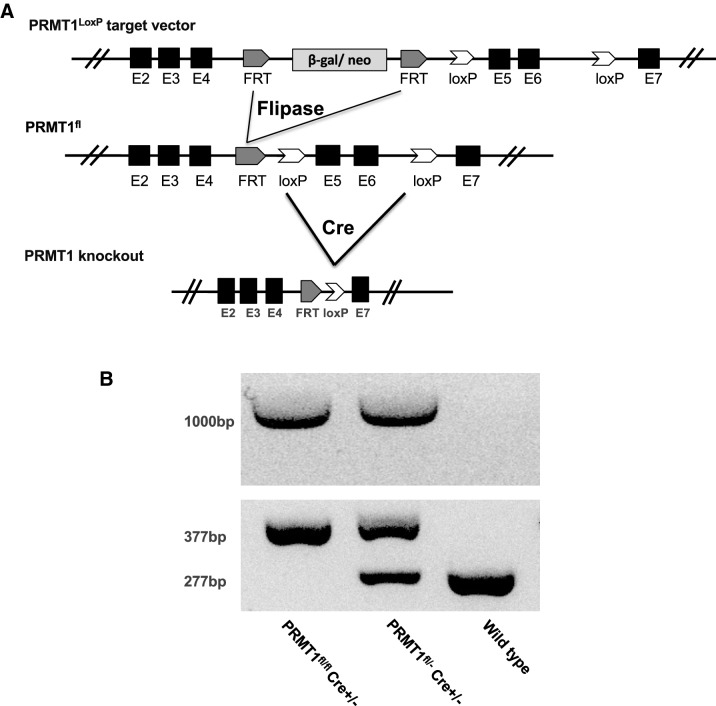
Generation and confirmation of intestinal epithelium-specific PRMT1 conditional knockout mice. **A** Schematic diagram of the wild-type PRMT1 allele with LoxP sites (white arrowheads) flanking exons 5 and 6, and an FRT-flanked β-galactosidase/neomycin (β-gal/neo) cassette, which can be removed by crossing the PRMT1^LoxP^ mice with Flp (Flipase)-expressing mice to produce mice containing PRMT1^fl^ allele. The resulting PRMT1^fl^-containing mice can be crossed with mice expressing the Cre recombinase under the control of the Villin promoter to remove the floxed exons 5 and 6, thus knocking out this PRMT1 allele. **B** PCR-genotyping of genomic DNA extracted from tail biopsies. Homozygous PRMT1^fl/fl^ mice were crossed with mice that were heterozygous in Villin Cre and floxed PRMT1 (PRMT1^fl/+^Cre^+/−^). The resulting animals as well as wild type (WT) were genotyped by PCR with a PRMT1 primer combination that distinguishes WT, PRMT1^fl/fl^ and PRMT1^fl/+^ genotypes, which produced bands in agarose gel of 377 bp for PRMT1 floxed allele and 277 bp for WT PRMT1 allele., respectively. The presence or absence of Cre was also analyzed by PCR with a specific primer pair to produce a band of about 1000 bp

Floxed PRMT1 mice containing the β-galactosidase/neomycin (β-gal/neo) cassette flanked by two FRT sites (Prmt1, MBBS; EPD0070_1_B01) (Fig. 1A) were obtained from Wellcome Trust Sanger Institute. Mice with enhanced FLP1 recombinase and the Villin-Cre transgenic mice were obtained from the Jackson Lab. All mice were maintained in accordance with the NIH animal facility guidelines for laboratory animal research. All animal care and treatments were done as approved by the Animal Use and Care Committee of Eunice Kennedy Shriver National Institute of Child Health and Human Development (NICHD), National Institutes of Health (NIH).

### Generation of intestinal specific PRMT1 knockout (KO) mice

The mice obtained from Wellcome Trust Sanger Institute were crossed with mice expressing the enhanced FLP1 recombinase (The Jackson Lab) to delete the β-gal/neo cassette via FRT sites recognized by FLP1 (Fig. 1A). The resulting floxed PRMT1 heterozygous male and female mice (PRMT1^fl/+^) mice were crossed with each other or with Villin-Cre transgenic mice (The Jackson Lab) to generate homozygous floxed PRMT1 mice (PRMT1^fl/fl^) or heterozygous floxed PRMT1 and Villin-Cre (PRMT1^fl/+^Cre^+/−^) mice. PRMT1^fl/fl^ and PRMT1^fl/+^Cre^+/−^ mice were crossed to produce PRMT1^fl/fl^Cre^+/−^ mice, which will have both PRMT1 alleles disrupted in the intestinal epithelium (Fig. 1A, B).

### Genotyping

Mouse tail tips were used to isolate genomic DNA with Quiagen DNeasy Blood & Tissue Kit Protocol. The DNA was used for PCR with forward primer 5′-GTGCTTGCCATACAAGAGATCC-3′ and reverse primer 5′-ACAGCCGAGTAGCAAGGAGG-3′ for PRMT1; and forward primer 5′-GTGTGGGACAGAGAACAAACC-3′ and reverse primer 5′-ACATCTTCAGGTTCTGCGGG for Cre. The PCR products were analyzed in a 2% agarose gel.

### Measurements for the small intestine, intestinal crypts, villi, and body

After euthanization, PRMT1^fl/fl^Vil-Cre^+/−^ and control (PRMT1^fl/fl^Vil-Cre^−/−^) mice were weighed, and the small intestine and colon were dissected and their lengths were measured. The length of the dissected small intestine was measured from the beginning of the duodenum to the end of the ileum. The length of the villus was measured from the mouth of the crypt to the tip of the villus on hematoxylin and eosin (H&E)-stained intestinal cross-sections, which were also used for crypt measurements.

### Histology processing and staining

The intestines were removed from age-matched wild type (PRMT1^fl/fl^Vil-Cre^−/−^) and knockout (PRMT1^fl/fl^Vil-Cre^+/−^) animals. The isolated intestine was flushed with ice-cold 1× phosphate-buffered saline (PBS) and fixed in 4% formaldehyde, followed by embedding in paraffin and then cutting into 5 µm sections. When indicated, the distal (within the second half of the ileum) and proximal (within the first half of the jejunum) sections of the small intestine were analyzed separately to see if there were regional differences in the small intestine.

For H&E staining, the 5 µm sections were stained with H&E solution following the manufacturer’s protocol (Sigma-Aldrich) and analyzed under a bright-field microscope.

For Alcian Blue staining, paraffin sections of the intestine were stained with an Alcian Blue Kit (Abcam). The blue Goblet cells in the crypt and villus were counted.

### EdU staining

Transit amplifying cells were labeled with 5-ethynyl-2′-deoxyuridine (EdU) staining method. Click-iT™ EdU Cell Proliferation Kit for Imaging (Thermo Fisher Scientific, Alexa Fluor™ 594 dye) was applied following the manufacturer’s protocol. Briefly, in proliferation assay, PRMT1^fl/fl^Vil-Cre^+/−^ and control mice were intraperitoneally injected with EdU (1 mg per mouse) 2 h before euthanization. In EdU pulse-chase assay, PRMT1^fl/fl^Vil-Cre^+/−^ and control mice were intraperitoneally injected with EdU (1 mg per mouse), and the mice were euthanized at 2 h, 24 h, or 48 h, after injection. The EdU-labeled intestine from PRMT1^fl/fl^Vil-Cre^+/−^ and control mice were isolated and the intestine paraffin sections (5 µm) were prepared as above.

The intestine paraffin sections were baked at 60 °C for 1 h followed by deparaffinized with xylene and rehydrated through a graded series of ethanol. Then the sections were incubated with Click-iT® Plus reaction cocktail for 30 min at room temperature, washed once with 1× PBST (1× PBS and 0.05% Tween-20), mount on glass slides with ProLong® Gold Antifade Mountant with DAPI (Thermo Fisher Scientific). The fluorescent pictures for different colors and different sections were taken under the same settings and then analyzed by using Fiji ImageJ at the same setting to count the positive cell numbers.

### TUNEL assay

Apoptotic cells were labeled with terminal deoxynucleotidyl transferase dUTP nick end labeling (TUNEL) method. In situ Cell Death Detection Kit (Roche, TMR red) was used for detection by following the manufacturer’s protocol. Briefly, 5 µm paraffin intestinal sections were baked at 60 °C for 1 h followed by deparaffinized with xylene and rehydrated through a graded series of ethanol. Antigen retrieval was performed by microwaving the sections (700 W; 5 min) in sodium citrate buffer (pH 6.0) followed by rinsing in PBS. The sections were incubated for 30 min in 0.1 M Tris-HCL, pH 7.5, containing 3% bovine serum albumin and 20% normal bovine serum at room temperature for blocking non-specific binding site, washed in PBS, and incubated with TUNEL reaction mixture at 37 °C for 1 h. After removing the TUNEL reaction mixture, sections were washed in PBS three times, and then mounted with ProLong® Gold Antifade Mountant with DAPI (Thermo Fisher Scientific). The fluorescent pictures of different sections were taken under the same settings and then analyzed by using Fiji ImageJ at the same setting to count the positive cell numbers.

### Immunohistochemistry

For immunofluorescent staining of PRMT1 or lysozyme in intestinal epithelial cells, paraffin sections (5 µm) were baked at 60 °C followed by dewaxing in xylene and rehydration through a series of different concentrations of ethanol. Antigen retrieval was performed by boiling in the antigen retrieve buffer (1 mM Tris–HCl, 1 mM EDTA, and 0.05% Tween-20) for 3 min at 125 °C followed by washing the slides three times in 1× PBST (1× PBS and 0.05% Tween-20) for 5 min each. After incubation in the blocking buffer (10% normal goat serum in PBS) for 1 h at room temperature, primary antibody (PRMT1 antibody from Upstate was diluted 1:100, lysozyme antibody from Dako was diluted 1:400) was added, and the slides were incubated at 4 °C overnight. The slides were then washed in 1× PBST and subsequently incubated with a fluorescence secondary antibody (Thermo Fisher Scientific) for 2 h at room temperature, washed three times with 1× PBST, then mount with ProLong® Gold Antifade Mountant with DAPI. For double-labeling PRMT1-positive cells and proliferating cells in intestinal cross sections from EdU-injected mice, the sections were first processed for PRMT1 immunostaining, then the slides were washed in 1× PBST for 5 min, followed by EdU staining. To co-stain PRMT1 and lysozyme, we optimized the incubation times as following: sections were incubated with PRMT1 antibody overnight and then with the first secondary antibody for 2 h. Subsequently, the slides were washed three times in 1× PBST for 5 min each, followed by incubation with lysozyme antibody overnight and then with the second secondary antibody just for 15 min. The fluorescent pictures of different sections were taken under the same settings. The positive cells were counted and analyzed by using Fuji Image J.

### In situ hybridization of intestinal stem cells

In situ hybridization with a RNAscope 2.5 HD Reagent Kit-Brown (322300; Advanced Cell Diagnostics) was performed on 5 µm formalin-fixed, paraffin-embedded sections according to the manufacturer’s instructions. The RNAscope probes used were Lgr5 (NM_010195.2, target region 2165–3082, cat no. 312171) or Olfm4 (NM_001030294.1, target region 25–1043, cat no. 311831), and the positive control probe Ppib (NM_011149.2, target region 98–856, cat no. 313911), and the negative control probe DapB (EF191515, target region 414–862, cat no. 310043).

### Statistical analysis

Differences between groups were analyzed for statistical significance by using a two-tailed student’s t-test. All experiments were repeated at least three times. Results are expressed as means ± standard deviation (SD) (P < 0.05). For the analysis of intestinal cross-sections, individual cross-sections instead of individual animals were used as samples for the Student’s t-test. All data are expressed as the mean–standard error of the mean.

## Results

### Intestinal epithelium-specific knockout of PRMT1 leads to altered adult intestinal structure

To overcome the embryonic lethal phenotype due to total PRMT1 knockout, we obtained a PRMT1 mouse line (PRMT1^LoxP^) from Wellcome Trust Sanger Institute. In this mouse line, a wild type PRMT1 allele has LoxP sites flanking exons 5 and 6, and an FRT-flanked β-galactosidase/neomycin (β-gal/neo) cassette, which can be removed by crossing the PRMT1^LoxP^ mice with Flp (Flipase)-expressing mice to produce mice containing PRMT1^fl^ allele (Fig. 1A). The PRMT1^fl^ mice can be crossed with mice expressing the Cre recombinase, which recognize the LoxP sites to remove the intervening sequence. In our study, we used a mouse line where the Cre was under the control of the Villin promoter (Vil-Cre mice) to remove the floxed exons 5 and 6, thus knocking out this PRMT1 allele, specifically in the intestinal epithelium (Fig. 1A). These different PRMT1 mutant alleles can be easily genotyped by PCR analyses of mouse genomic DNA (Fig. 1B). Importantly, by inter-crossing different mouse lines, we were able to obtain mice containing both PRMT1 alleles with floxed exons 5 and 6 and also Villin-Cre (PRMT1^fl/fl^Cre^+/−^), in which the exons 5 and 6 would be removed by Cre in both PRMT1 alleles in the intestinal epithelium.

**Fig. 2:**
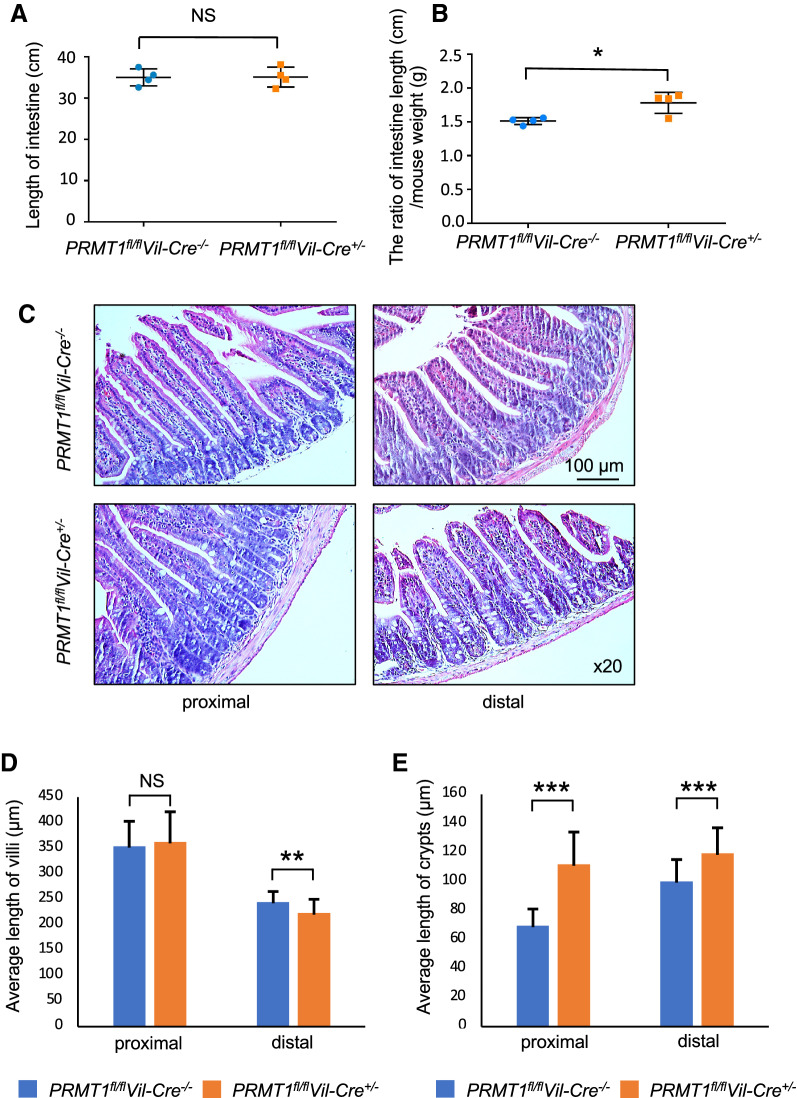
Intestinal epithelium-specific deletion of PRMT1 alters the structural characteristics of adult intestine. **A** PRMT1 knockout does not affect absolute intestinal length. The length of the intestine was measured for 8-week-old adult wild type (*PRMT1*^*fl/fl*^*Vil-Cre*^*−/−*^) or intestinal epithelium-specific PRMT1 knockout (*PRMT1*^*fl/fl*^*Vil-Cre*^+/−^) mice. **B** PRMT1 knockout increases the relative length of the intestine normalized by body weight. The length of the intestine (cm) as in A was normalized against the body weight of the animals (grams). **C**–**E** PRMT1 knockout increases the crypt length. The intestine from 8-week-old wild type or PRMT1 knockout mice were dissected and stained with H&E staining (**C**) and the length of the villus (**D**) or crypt (**E**) were measured for both distal and proximal small intestine from multiple sections per animal. Note that the crypt length was increased in both distal and proximal small intestine. The villus length was not altered in the proximal small intestine and slightly decreased in the distal small intestine. For both PRMT1 knockout and control littermates, n = 4. *p < 0.05, **p < 0.01, ***p < 0.001. *ns* no significant

The mice with homozygous intestinal epithelium-specific knockout of PRMT1 (PRMT1^fl/fl^Cre^+/−^) as generated above developed to adulthood with normal appearance (not shown). To determine the effect of the knockout on the intestine, we analyzed the intestine of 8 weeks, 13 weeks, or 5 months old control (PRMT1^fl/fl^Cre^−/−^) (referred to as wild type) and intestinal epithelium-specific PRMT1 knockout (i.e., PRMT1^fl/fl^Cre^+/−^, and simply referred to as PRMT1 knockout in the rest of the paper) mice (note that as the findings were similar in the three age groups, we only presented data for one age group). We first measured small intestine length and found it to be similar between wild type and PRMT1 knockout animals (Fig. 2A). Interestingly, we noticed that the knockout animals appeared to be slightly smaller and thus determined the relative intestinal length by normalizing against the body weight. The relative length of the small intestine was slightly longer in the PRMT1 knockout mice (Fig. 2B).

The adult small intestine consists of villus-crypt units (Fig. 2C) with the rapidly proliferating transit amplifying cells and stem cells localized in the crypt while the most abundant differentiated epithelial cells, the absorptive epithelial cells, localized on the villus. When we analyzed the cross-section of the small intestine and measure the lengths of the villi and crypts, we observed that PRMT1 knockout had no effect on the length of the villi in the proximal small intestine and a slight reduction in the length of the villi in the distal small intestine (Fig. 2D). On the other hand, the knockout caused a significant increase in the length (or depth) of crypts, with a bigger effect on the proximal small intestine (Fig. 2E).

### The number of crypt base stem cells are not affected by the deletion of PRMT1 from the intestinal epithelium

**Fig. 3:**
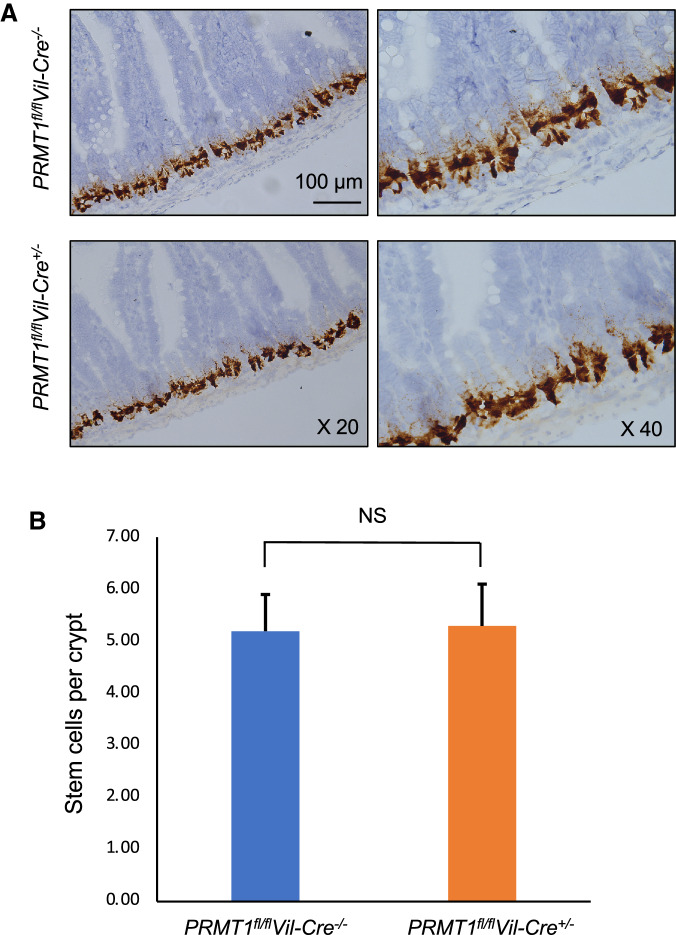
PRMT1 knockout does not affect intestinal stem cell number. The proximal small intestine of 13-week-old adult wild type (*PRMT1*^*fl/fl*^*Vil-Cre*^*−/−*^) or intestinal epithelium-specific PRMT1 knockout (*PRMT1*^*fl/fl*^*Vil-Cre*^+/−^) mice were sectioned and analyzed by in situ hybridization with Olfm4 probe for crypt base stem cells (**A**). The number of stem cells were counted visually from multiple sections per animal (**B**). For both PRMT1 knockout and wild type littermates, n = 4. *p < 0.05, **p < 0.01

Earlier studies on the role of PRMT1 during intestinal metamorphosis in the anuran *Xenopus laevis* has implicated an important role of PRMT1 in the development of adult intestinal stem cells during metamorphosis and this role may be conserved in mouse based on PRMT1 expression during perinatal intestinal development [31]. Given the structural changes in the crypt of intestinal epithelium-specific PRMT1 knockout mice, we speculated that the PRMT1 knockout might affect adult intestinal stem cells in adult mice. Thus, we analyzed the stem cells in the proximal small intestine by using in situ hybridization with antisense mRNA probes for Olfm4 (Fig. 3) and Lgr5 (Additional file 1: Fig. S1), two well-known markers for crypt base stem cells. As expected, both markers were detected in the crypt base cells (Fig. 3A and Additional file 1: Fig. S1A). Quantification showed that the signals were similar between the wild type and PRMT1 knockout intestine (Fig. 3B and Additional file 1: Fig. S1B), indicating that PRMT1 knockout does not affect the number of crypt base stem cells in the adult intestine.

### PRMT1 knockout increases cell proliferation in the crypt

**Fig. 4:**
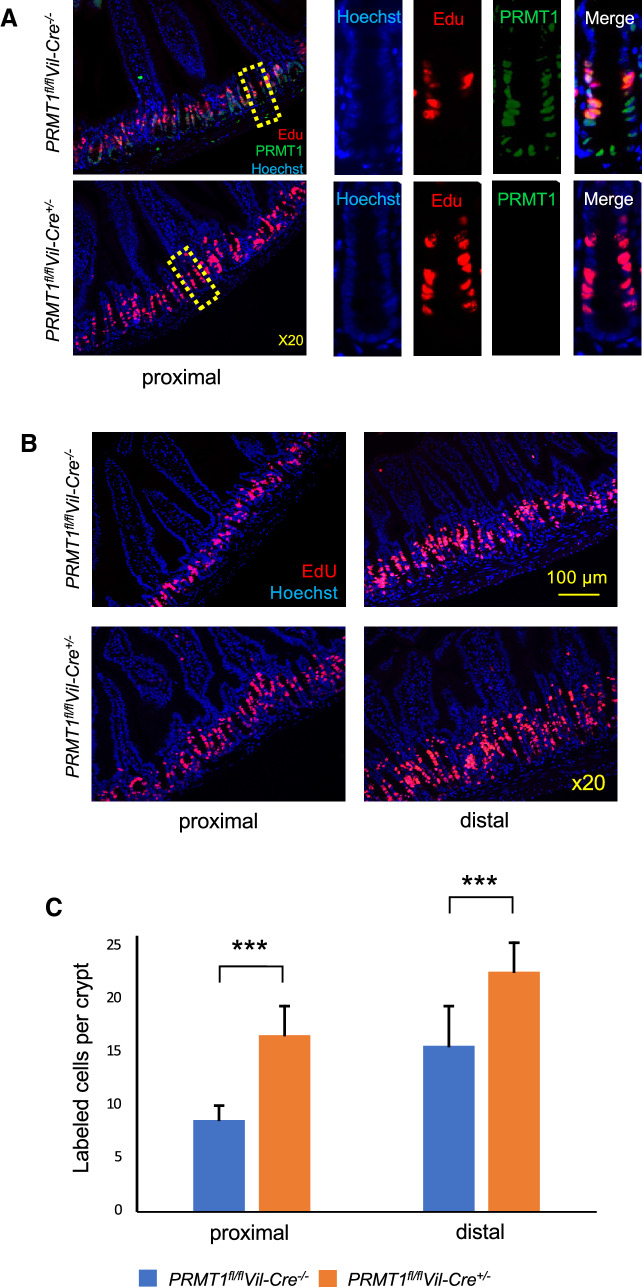
Intestinal epithelium-specific deletion of PRMT1 increases cell proliferation in the crypt. **A** High levels of PRMT1 are present in the crypt base and transit amplifying cells in wild type small intestine. 8-week-old adult wild type (*PRMT1*^*fl/fl*^*Vil-Cre*^*−/−*^) or PRMT1 knockout (*PRMT1*^*fl/fl*^*Vil-Cre*^+/−^) mice were injected with EdU to label proliferating cells. The proximal small intestine was then isolated for detection of EdU and PRMT1 expression by immunohistochemistry. The DNA was stained with Hoechst. Note that EdU labeled mostly transit amplifying cells which had high levels of PRMT1 in the wild type animals. There were also PRMT1-expressing cells at the bottom of the crypt where stem cells reside. Expectedly, no PRMT1 proteins were found in the epithelium of PRMT1 knockout mice (*PRMT1*^*fl/fl*^*Vil-Cre*^+/−^). Note the increased cell proliferation in the crypts of PRMT1 knockout mice. **B**, **C** PRMT1 knockout increases cell proliferation in both proximal and distal small intestine. Both proximal and distal small intestine of wild type and PRMT1 knockout mice were analyzed by EdU labeling and Hoechst staining (**B**) and the number of proliferating transit amplifying cells were quantified from multiple sections per animal (**C**). For both PRMT1 knockout and control wild type littermates, n = 4. ***p < 0.001

Other than the crypt base stem cells, there are three major cell types in the intestinal crypt: transit amplifying cells, Paneth cells, and Goblet cells. The altered crypt structure suggest changes in one or more of these cell types. We first analyzed cell proliferation in the crypts of adult mice by injecting EdU into mice to label proliferating cells. As shown in Fig. 4A, strong EdU labeling was present in the middle of the crypts where transit amplifying cells are located in the wild type animals, as expected. Interestingly, immunohistochemical analysis of PRMT1 expression showed that PRMT1 was highly expressed in the middle and bottom of the crypts and that the EdU-labeled cells had high levels of PRMT1 in the wild type animals, indicating that PRMT1 is expressed in the transit amplifying cells and stem cells located in the crypt base. In the PRMT1 knockout mice (*PRMT1*^*fl/fl*^*Vil-Cre*^+/−^), there was expectedly, no PRMT1 expression detected in the intestinal epithelium (Fig. 4A). Importantly, PRMT1 knockout appeared to have increased cell proliferation in the crypt (Fig. 4A). To further analyze the effect of the PRMT1 knockout on cell proliferation, we analyzed cell proliferation in both proximal and distal small intestine (Fig. 4B). Quantitative analysis showed that PRMT1 knockout increased cell proliferation in both proximal and distal small intestine (Fig. 4C). Given the known role of PRMT1 as a TR coactivator and known effect of T3 on the intestine, we had expected that PRMT1 knockout might reduce intestinal stem cells and/or inhibit cell proliferation in the crypt. Thus, our finding here is surprising and suggest that either PRMT1 functions mainly through TR-independent pathway(s) to affect cell proliferation or that PRMT1 knockout in only the epithelium but not the other intestinal tissues, alters cell–cell interactions such that the cell proliferation is increased despite reduced TR signaling due to the knockout.

### Intestinal epithelium-specific deletion of PRMT1 alters cell differentiation in the crypt

**Fig. 5:**
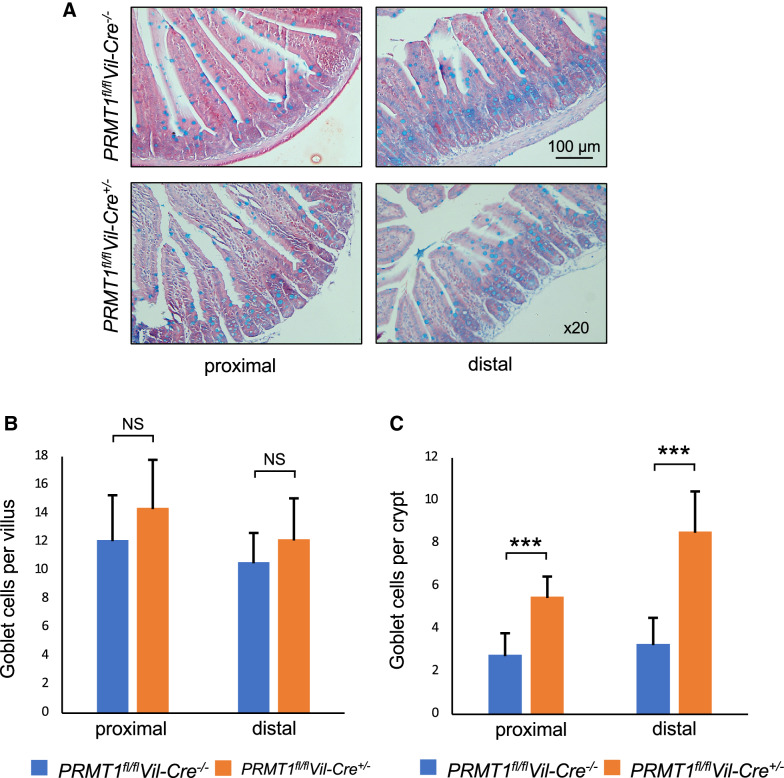
Intestinal epithelium-specific deletion of PRMT1 increases Goblet cells in the crypt. The proximal and distal small intestine of 8-week-old adult wild type (*PRMT1*^*fl/fl*^*Vil-Cre*^*−/−*^) or PRMT1 knockout (*PRMT1*^*fl/fl*^*Vil-Cre*^+/−^) mice were sectioned and stained with Alcian Blue to detect the goblet cells (**A**). The number of Goblet cells in the villi and crypts were counted from multiple sections per animal and graphed (**B**, **C**). Note that Goblet cell number was increased in the crypt but not villus of both distal and proximal intestine in PRMT1 knockout mice. For both PRMT1 knockout and control wild type littermates, n = 4. ***p < 0.001. *ns* no significant

We next investigated if PRMT1 knockout in the intestinal epithelium affected the other two major cell types in the crypt: Goblet and Paneth cells. We first stained cross-sections of the proximal and distal small intestine of 8-week-old adult wild type (*PRMT1*^*fl/fl*^*Vil-Cre*^*−/−*^) or PRMT1 knockout (*PRMT1*^*fl/fl*^*Vil-Cre*^+/−^) mice with Alcian Blue to detect the Goblet cells (Fig. 5A). Quantitative analyses showed that the number of Goblet cells in the villi were similar between the wild type and knockout mice for both proximal and distal small intestine (Fig. 5B). On the other hand, the number of Goblet cells were increased in the crypt for both distal and proximal intestine in PRMT1 knockout mice.

**Fig. 6:**
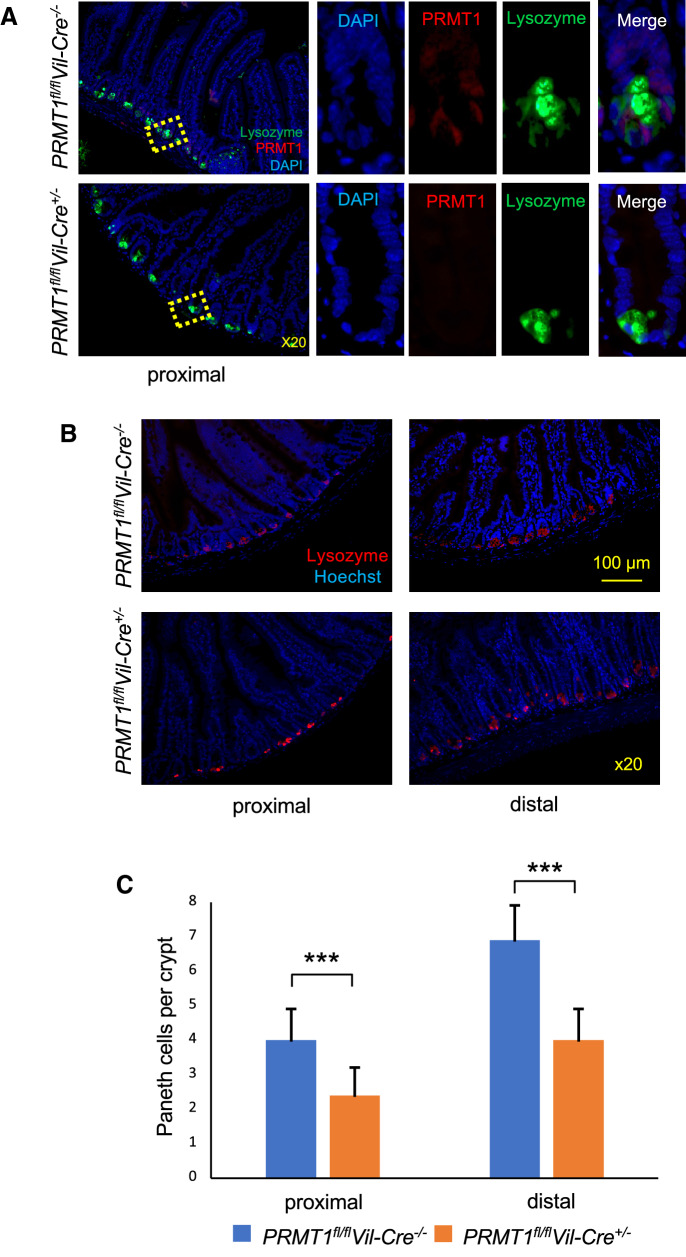
Intestinal epithelium-specific deletion of PRMT1 reduces Paneth cells in the crypt. **A** PRMT1 protein is low or absent in Paneth cells in the adult wild type intestine. The small intestine of 8-week-old adult wild type (*PRMT1*^*fl/fl*^*Vil-Cre*^*−/−*^) or PRMT1 knockout (*PRMT1*^*fl/fl*^*Vil-Cre*^+/−^) mice were sectioned and stained with anti-PRMT1 antibody for PRMT1 expression (red) or anti-lysozyme antibody for Paneth cells in the crypt (green). Note that the signals for PRMT1 and lysozyme were in different cells in the wild type intestine, with PRMT1-expressing cells surrounding the lysozyme-positive Paneth cells, which is consistent with PRMT1-expressing cells being the crypt base stem cells). Expectedly, PRMT1 was not detected in the knockout epithelium. **B**, **C** PRMT1 knockout reduces Paneth cells in both proximal and distal small intestine. The proximal and distal small intestine of wild type and PRMT1 knockout mice were stained with anti-lysozyme antibody for Paneth cells and Hoechst for DNA (**B**) and the number of Paneth cells were quantified from multiple sections per animal (**C**). For both PRMT1 knockout and control wild type littermates, n = 4. ***p < 0.001. *ns* no significant

We then detected the Paneth cells in the small intestine with anti-lysozyme antibody and found that the Paneth cells in the crypt base were distinct from the cells expressing high levels of PRMT1 protein as detected by anti-PRMT1 antibody immunohistochemistry (Fig. 6A), supporting that the PRMT1-expressing crypt base cells are adult stem cells. Furthermore, when Paneth cells in both proximal and distal small intestine were analyzed, we found that PRMT1 knockout led to a significant reduction of Paneth cells in both regions (Fig. 6B, C). Thus, PRMT1 knockout changes both cell proliferation and differentiation in the crypt along the entire small intestine.

### Cell death in the intestine is increased in PRMT1 knockout intestine

**Fig. 7:**
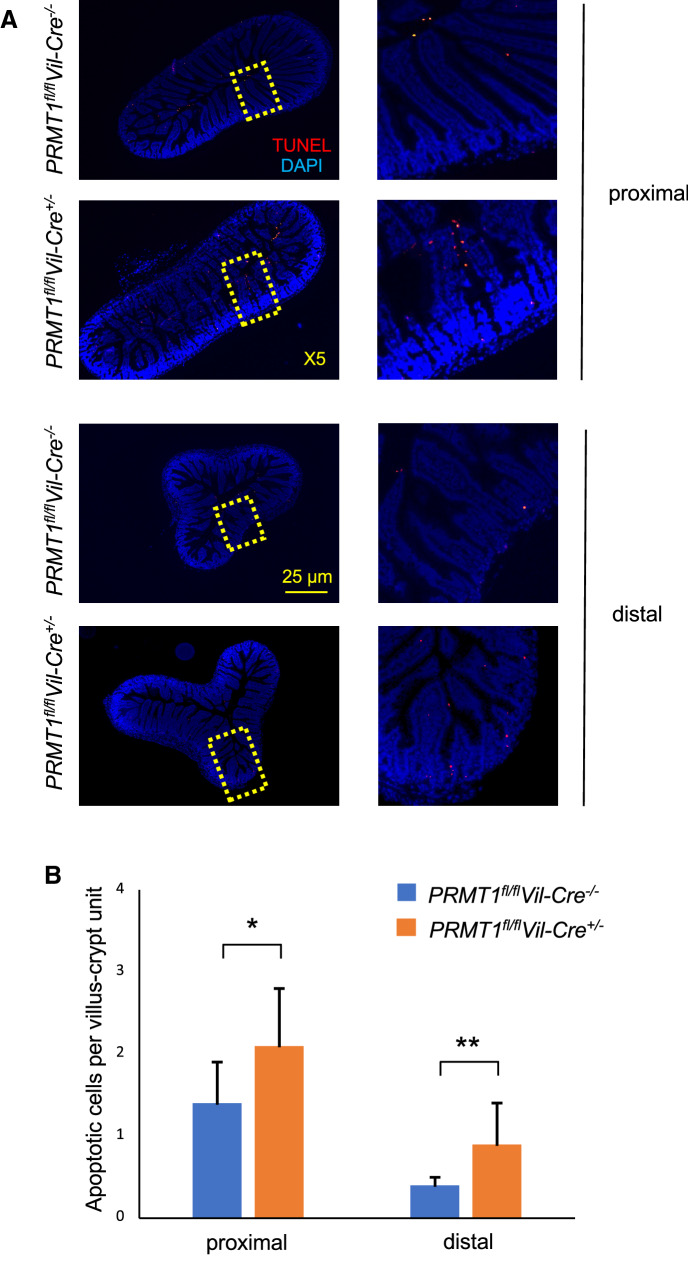
PRMT1 knockout increases cell death in the intestine. The proximal and distal small intestine of 8-week-old adult wild type (*PRMT1*^*fl/fl*^*Vil-Cre*^*−/−*^) or intestinal epithelium-specific PRMT1 knockout (*PRMT1*^*fl/fl*^*Vil-Cre*^+/−^) mice were sectioned and analyzed with TUNEL for apoptotic cells (red) and DAPI for DNA (blue) (**A**). The apoptotic cells per villus-crypt unit were quantified from multiple sections per animal (**B**). For both PRMT1 knockout and control wild type littermates, n = 4. *p < 0.05, **p < 0.01

The intestinal epithelium in the adult mice is constantly self-renewed through cell proliferation in the crypt and cell removal via apoptosis, mainly at or near the tip of the villus. The increased cell proliferation in the crypt of PRMT1 knockout mice in the absence of significant change in the villus size and morphology suggests a likely compensatory increase in cell death on the villus. To test this possibility, we next investigated the effect of PRMT1 knockout on apoptosis in the intestine. Cross-sections of the proximal and distal small intestine of 8-week-old adult wild type and PRMT1 knockout mice were analyzed with TUNEL staining for apoptotic cells (Fig. 7A). Quantitative analysis showed that the apoptotic cells were significantly increased in both the proximal and distal intestine of PRMT1 knockout mice (Fig. 7B), supporting a model of epithelial homeostasis through compensatory changes in cell proliferation in the crypt and cell death on the villus [43].

### PRMT1 knockout accelerates epithelial migration and turnover in the intestine

**Fig. 8:**
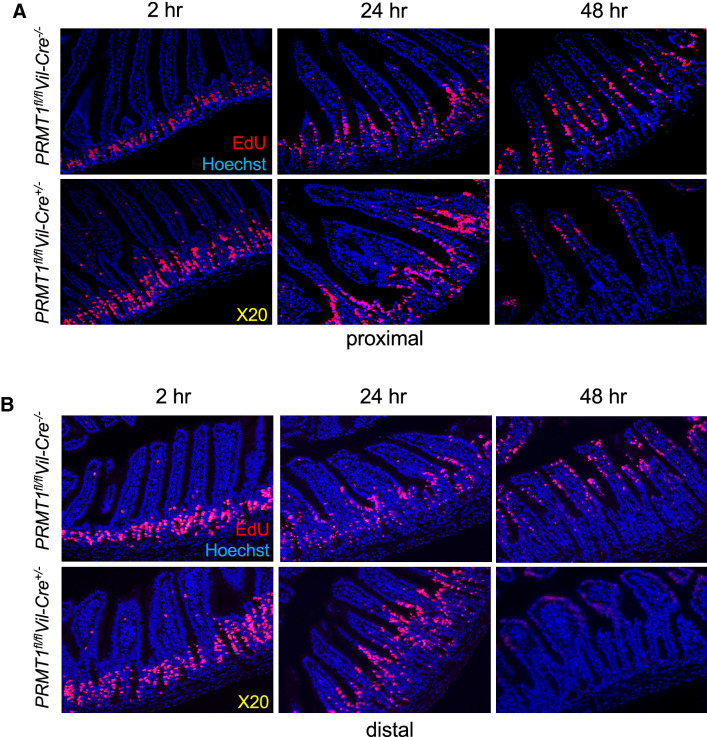
Intestinal epithelium-specific deletion of PRMT1 accelerates the epithelial migration along the crypt-vilus axis. The proximal (**A**) or distal (**B**) small intestine of 8-week-old wild type (*PRMT1*^*fl/fl*^*Vil-Cre*^*−/−*^) or PRMT1 knockout (*PRMT1*^*fl/fl*^*Vil-Cre*^+/−^) mice were sectioned at indicated times after EdU injection. Note that in both proximal and distal intestine, essentially all EdU-labeled cells were located in the crypts at 2 h after EdU injection in both wild type and knockout animals. 24 h after EdU injection, EdU-labeled cells migrated up to the lower middle of villus, with the faster migration in the knockout mice. 48 h after EdU injection, EdU-labeled cells migrated to the middle (for proximal intestine) or near the top (for distal intestine) of villus in wild type animals but in the knockout animals, most of the labeled cells were lost, suggesting that they had migrated to the very top of the villus and undergone apoptosis

The intestinal epithelial self-renewal occurs through cell proliferation in the crypt, mainly the transit amplifying cells, followed by their migration along the crypt-villus axis as they differentiate, before eventual death mostly near the tip of the villus. The increased cell proliferation and apoptosis in the epithelium of PRMT1 knockout mice suggest that PRMT1 regulates epithelial cell migration and turnover. To investigate this, we transiently labeled proliferating cells by injecting EdU into 8-week-old wild type (*PRMT1*^*fl/fl*^*Vil-Cre*^*−/−*^) or knockout (*PRMT1*^*fl/fl*^*Vil-Cre*^+/−^) mice and isolated the proximal and distal small intestine at 2, 24, or 48 h after EdU injection. As expected, at 2 h after EdU injection, essentially all EdU-labeled cells were located in the crypts in both wild type and knockout animals (Fig. 8). By 24 h, most EdU-labeled cells migrated up to the lower middle of villi of both proximal and distal small intestine in the wild type mice. Interestingly, in the knockout mice, the labeled cells migrated further up on the villi, suggesting faster migration. 48 h after EdU injection, EdU-labeled cells migrated to the middle (for proximal intestine) or near the top (for distal intestine) of villi in wild type animals (Fig. 8). Interestingly, much fewer labeled cells were found in either proximal or distal small intestine of the PRMT1 knockout mice and these cells were at or near the tips of the villi. These results suggest that most of the labeled cells had migrated to the very top of the villi and undergone apoptosis in the knockout mice. Thus, PRMT1 knockout led to faster migration of the epithelial cells and an increased turnover rate.

## Discussion and conclusion

The mouse intestine has long been studied as a model for tissue repair and regeneration as well as tumor development due to the constant self-renewal via the proliferation of the adult stem cells in the crypts throughout adult life. The formation of the adult stem cells in various organs during vertebrate development is relatively poorly understood but the underlying molecular pathways likely provide important clues for different stem cell-related pathological conditions such as cancer. The discovery that expressing certain transcription factors leads to the formation of induced pluripotent stem cells demonstrated the importance of transcription factors in determining stem cell identity [44–47]. Our studies here indicate that PRMT1, a histone methyltransferase-encoding transcription cofactor for TR and other nuclear hormone receptors [24, 25, 48], plays a critical role in regulating cell proliferation and differentiation in the intestinal crypt.

The intestinal epithelial specific knockout of PRMT1 leads to elongated intestinal crypts in the adult mice. As most of the cells in the crypt are transit-amplifying cells, it is not surprising that PRMT1 knockout affected cell proliferation. In the wild type intestine, the proliferation marker clearly labeled the cells in the zone of the transit-amplifying cells that expressed high levels of PRMT1 and they were significantly increased upon removing PRMT1 from the intestinal epithelium, indicating that PRMT1 is required for the intestine to mature into and/or maintain the proper adult crypt morphology but also plays an inhibitory role in the proliferation of these transit-amplifying cells in the adult intestine. Additionally, the knockout animals also had reduced Paneth cells and increased Goblet cells in the crypt.

The mouse intestine undergoes a biphasic development, the initial formation of a neonatal intestine followed by the maturation into the adult form after birth when plasma T3 concentration peaks around 2–3 weeks later in mouse [5, 8, 31, 49, 50]. In addition, T3 deficiency or TR mutations/knockout leads to reduced cell proliferation in the adult mouse intestine [9–14]. In particular, we have shown recently that the adult intestine in a mutant mouse expressing a knockin dominant negative TR, which inhibits T3-signaling, has reduced cell proliferation [14]. Furthermore, PRMT1 is highly expressed in the transit amplifying cells in the crypt. All these suggest that knocking out PRMT1, a coactivator for liganded TR, in the intestinal epithelium should reduce T3-signaling, leading to decreased cell proliferation in the crypt, contrary to what we observed here. Thus, while it is possible that PRMT1 can enhance the function of TR to facility cell proliferation in the crypt, it has a more dominant role, through a yet-unknown but likely TR-independent pathway(s), to inhibit cell proliferation in the adult intestine.

The development of the intestine is conserved in vertebrates. There is also a biphasic intestinal development in anurans such as *Xenopus laevis*. The tadpole intestine consists of predominantly a monolayer of larval epithelial cells, surrounded by thin connective tissue and muscle layers, resembling the intestine in newborn mice [5, 32, 49, 50]. During metamorphosis, the larval epithelial cells undergo apoptosis and concurrently, adult epithelial stem cells are formed de novo through the dedifferentiation of some larval epithelial cells via yet known mechanism [32, 51, 52]. Toward the end of metamorphosis, the adult epithelial cells differentiate to establish a trough-crest axis of epithelial fold, resembling the crypt-villus axis in adult mammalian intestine [5, 32, 49, 50]. Interestingly, PRMT1 expression is upregulated during intestinal metamorphosis and PRMT1 is recruited by liganded TR to target genes [48]. More importantly, transgenic overexpression of PRMT1 increases the number of proliferating adult stem cells in the intestine during metamorphosis while knocking down endogenous PRMT1 expression reduces them. These findings led to the conclusion that PRMT1 is critical for T3-induced formation and/or proliferation of adult intestinal stem cells during amphibian metamorphosis [31]. Interestingly, PRMT1 expression is also upregulated during adult intestinal maturation in mouse and zebrafish when plasma T3 levels are high [31]. Thus, PRMT1 may have a conserved role in T3-dependent formation of the adult intestine in vertebrates. While such a role appears to contradict with our observation on the PRMT1 knockout adult intestine, it is possible that PRMT1 have two distinct roles in the intestine, one for the initial development of the adult intestine and another for the function and maintenance of the adult intestine. It will be of interest to study if and how PRMT1 knockout affect intestinal maturation during the neonatal period in mouse.

The PRMT1 knockout in the intestinal epithelium also altered cell differentiation in the crypt, leading to increased Goblet cells and reduced Paneth cells. Interestingly, PRMT1 expression is low or non-detectable by immunohistochemistry in these differentiated cells. Thus, it is likely, knocking out PRMT1 in the proliferating transit amplifying cells may impact their subsequent differentiation. It is also possible that PRMT1-deficient stem/transit amplifying cells may signal cells that do not express PRMT1 by altering cell–cell or cell-ECM interactions. As an arginine methyltransferase, PRMT1 can methylate histones and other proteins, to affect cellular processes other than transcription. For example, PRMT1 has been shown to be critical for canonical Wnt signaling and endolysosomal trafficking through cytoplasmic arginine methylation [53, 54]. Thus, PRMT1 may affect cell fate through mechanisms independent of its transcriptional cofactor activity, an area clearly of importance.

Despite the significant changes in the intestinal crypts, particularly the increased cell proliferation, caused by the epithelium-specific knockout of PRMT1, there is little effect on intestinal villi, where most of the intestinal cells are located. This appears to be due to a compensatory increase in cell death in the villus in the PRMT1 knockout intestine. Furthermore, in the knockout animals, the epithelial cells migrate faster along the crypt-villus axis and undergo faster turnover. We have earlier proposed that intestinal homeostasis involves a communication between the proliferating cells in the crypt and dying cells, mostly near the tip of the villus, through a mechanical and/or cell-ECM (extracellular matrix) interactions [43]. That is, increased cell proliferation in the crypt leads to more/faster upward migration of the differentiating epithelial cells. This increased upward migration can quickly exert an increased force on the epithelial cells in the entire epithelium through cell–cell or cell-ECM contacts in this monolayer tissue. In addition, it may force some of the differentiated epithelial cells to detach from the basement membrane, which is known to cause apoptosis in many types of epithelial cells [55]. Regardless of the exact mechanism, the simultaneous increase in villus cell death and crypt cell proliferation enable the PRMT1 knockout intestine to maintain an apparently normal villus morphology. On the other hand, one would expect that such increases in cell death and cell proliferation would results in increased epithelial replacement and regeneration in the knockout intestine. Furthermore, one splicing variant of PRMT1 is known to be expressed at higher levels in colon cancer tissues compared to normal tissues [56, 57], suggesting a role of PRMT1 in colon cancer. It would be interesting to investigate whether intestinal PRMT1 knockout influences such physiological and pathological events and if the effects are dependent on the methyltransferase activity of PRMT1 through pharmacological inhibition with PRMT1 inhibitors.

## Supplementary Information

**Additional file 1: Figure S1.** PRMT1 knockout does not affect the expression of adult stem cell marker Lgr5.

## Data Availability

Not applicable.
