# Supplementary material for: Protein arginine methyltransferase 1 regulates cell proliferation and differentiation in adult mouse adult intestine
Source: Cell Biosci. 2021 Jun 22;11:113. doi: 10.1186/s13578-021-00627-z (PMC8220849; doi:10.1186/s13578-021-00627-z)
Supplement: Supplementary file 1 — Additional file 1: Figure S1. PRMT1 knockout does not affect the expression of adult stem cell marker Lgr5. [file 13578_2021_627_MOESM1_ESM.pdf]

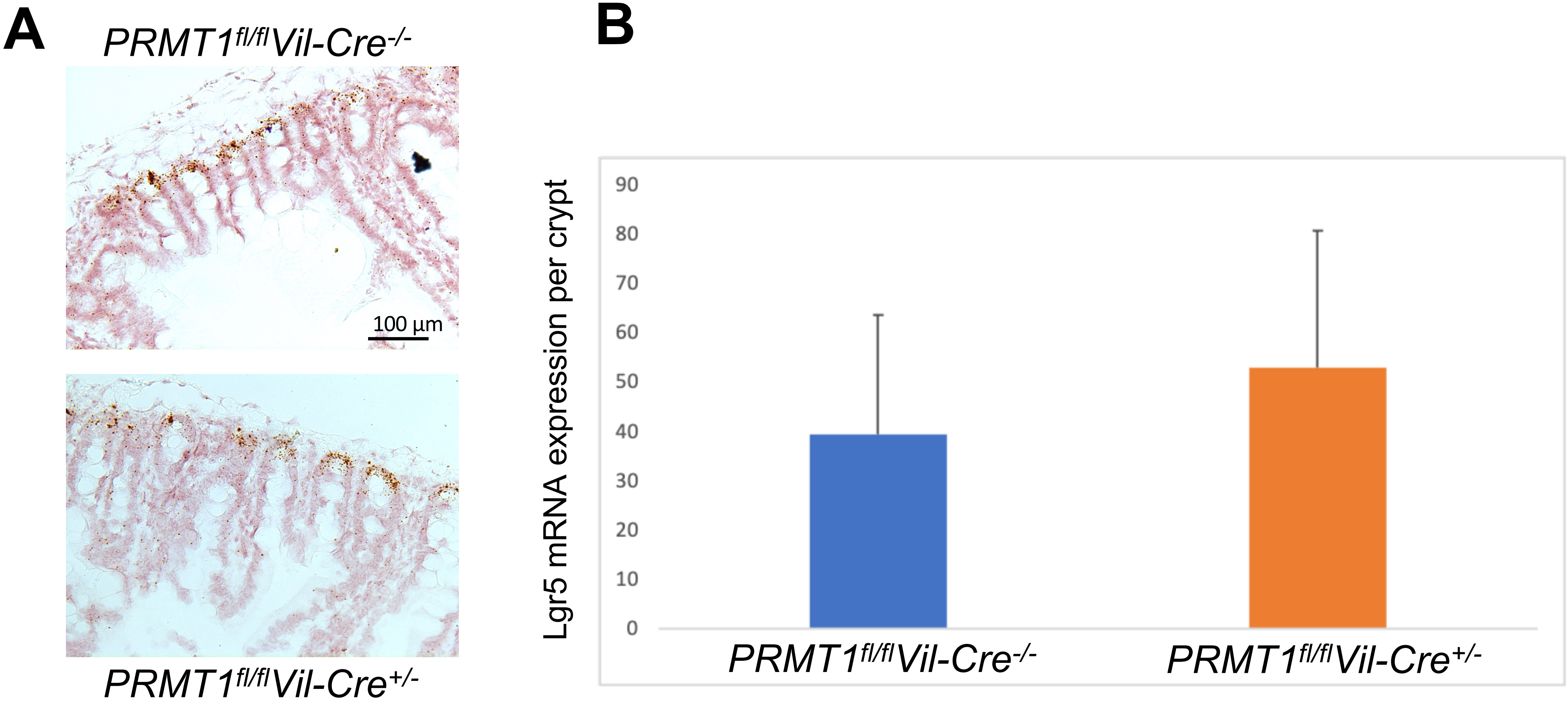

Figure S1. PRMT1 knockout does not affect the expression of adult stem cell marker Lgr5.

The proximal small intestine of 13-week-old adult wild type (*PRMT1*$^{fl/fl}$*Vil-Cre*$^{-/-}$) or intestinal epithelium-specific PRMT1 knockout (*PRMT1*$^{fl/fl}$*Vil-Cre*$^{+/-}$) mice were sectioned and analyzed by *in situ* hybridization with Lgr5 probe for crypt base stem cells (A). The Lgr5 signal in crypt was quantified from multiple sections per animal by using Imaris (B). For both PRMT1 knockout and wild type littermates, n=4.
